# Special Report on Standards for Radioactivity: Report on the 1989 Meeting of the Radionuclide Measurements Section of the Consultative Committee on Standards for the Measurement of Ionizing Radiations

**DOI:** 10.6028/jres.094.036

**Published:** 1989

**Authors:** Dale D. Hoppes

**Affiliations:** National Institute of Standards and Technology, Gaithersburg, MD 20899

**Keywords:** becquerel, comparisons of activity measurements, ^109^Cd, Consultative Committee on Standards for the Measurement of Ionizing Radiations, ^125^I, International Reference System for radionuclides, radioactivity standards, ^75^Se

## Abstract

This report describes the activities discussed at the 10th meeting of Section II of the Consultative Committee on Standards for the Measurement of Ionizing Radiations (Comité Consultatif pour les Etalons de Mesure des Rayonnements Ionisants, CCEMRI) held in May 1989 at Sèvres (France). Topics included present and future international comparisons of activity measurements, the status and possible extension of the International reference system for activity measurements of gamma-ray emitting nuclides, reports from other working groups, accomplishments at the International Bureau of Weights and Measures (Bureau International des Poids et Mesures, BIPM), and progress at member laboratories.

## 1. Background

One of the consultative committees of the International Committee of Weights and Measures (Comité International des Poids et Mesures, CIPM) which guides and assists the International Bureau of Weights and Measures (Bureau International des Poids et Mesures, BIPM) in ensuring the uniformity of physical measurements is the Consultative Committee on Standards for the Measurement of Ionizing Radiations (Comité Consultatif pour les Etalons de Mesure des Rayonnements Ionisants, CCEMRI) [1]. The variety of measurement types in the field led to the committee being set up with three sections. Section II, with the responsibility for radioactivity measurements, met on March 29–31, 198, with selected representatives from 11 countries and the European community laboratory participating.

The challenge in radioactivity standards is as much quantity as quality. Although the accuracy required and achieved for specifying the activity of a sample of a radionuclide is usually only of one percent, a standard must be developed for *each* pertinent radionuclide. Currently the National Institute of Standards and Technology has received justified requests for over 130 radionuclides, for example [2]. There is no official international standard for any radionuclide; rather, the standardizing laboratory in each country develops an appropriate measuring method which would allow a sample of the particular radionuclide to be measured with a known uncertainty.

Almost all countries have adopted the International System (SI) unit, the becquerel (Bq), specifying the activity of a sample in decays per second (s^−1^). Goals of the Radionuclides Section of CCEMRI are to improve measuring methods and better define their uncertainties, to compare results obtained in different countries, and to bring these results into congruence. The 10th meeting made considerable progress towards these goals.

## 2. Section Activities

The agenda of the meeting emphasized actions coordinated through the BIPM, the accomplishments of working groups, and the significant activities of the small BIPM group.

### 2.1 Comparisons of Activity Measurement

One mechanism with which national standardizing laboratories can compare results for a radionuclide is through simultaneous measurements of quantitative mass aliquants of a common solution. Twenty-eight such comparisons have been completed since 1961. The status of the three following current exercises was reported.

A 1986 comparison of ^109^Cd had been reported at the previous session in 1987, with satisfactory agreement on the activity [3]. However, 10 of the 18 laboratories participating also measured the 88-keV gamma-ray emission rate, which is the desired quantity when the radionuclide is used to calibrate the counting efficiency of gamma-ray spectrometry systems at that energy. It was pointed out that the combined result probably offers the most reliable measurement of the gamma-ray probability per decay for this radiation. The value obtained was *P_γ_*=0.03614±0.00012, where the uncertainty is the combined standard deviation of the means for activity and emission rate. It was recognized at the session that simply quoting this uncertainty might lead to an unrealistically small value being carried forth in evaluations, for uncertainties common to all measurements had not been considered. The BIPM staff was given the challenging task of making a realistic appraisal of the uncertainty before preparing an article describing the comparison for publication. This exercise emphasizes that nuclear data can be significant in the application of radionuclidic standards, that cooperative measurements can provide an incidental dividend of good nuclear data, and that standardizing laboratories should lead the way in demonstrating meaningful analyses of the uncertainties of such data.

The comparison in 1988 of solutions of ^125^I [4] also led to session reports of the measurements of another important nuclear datum, the half life, by 5 of the 19 laboratories participating. The value suggested for the comparison, (59.4±0.5) d, perhaps carried such a large uncertainty because of chemical instability or detection problems for the low-energy radiations in past measurements. These factors also can cause difficulties in activity measurements, as demonstrated by one laboratory in the comparison. Three ingenious methods gave very consistent results in-house with small estimated uncertainties—but were conspicuously 1.5% deviant from the average of measurement results from other laboratories. Sources had been prepared from a dilution of the solution supplied; a subsequent recheck with the main solution produced a result in agreement with the average of other participants. One important function of the comparisons is to alert all laboratories to potential problems; another is to test the variations observed against the combined uncertainties explicitly given in detail.

The third comparison discussed was that of ^75^Se, a radionuclide with a 16-ms delayed state in the daughter ^75^As. This radionuclide had been distributed only to five members of a working group for a “trial” comparison, a precaution which has revealed unexpected problems in some past comparisons. Only one result was reported at the session due to a delay in distributing the solution because of a high radionuclidic impurity level in the first material received from the commercial supplier. While impurities must always be considered in activity measurements, some effort is made to minimize the possibility of their beclouding the accuracy of the basic direct methods being compared.

As the above three examples show, comparisons are made only of radionuclides selected because of some special challenge. The same was true of those considered as possible candidates for future comparisons, once the “Se exercise is completed. “Tc emits only beta particles and has a half life sufficiently long that the thin solid sources required for some methods must be of low activity; ^210^Pb may have chemical problems; ^144^Ce—^144^Pr has shown discrepancies in measurements with different ionization chambers, perhaps because of the proportion of response due to bremsstrahlung; ^147^Pm is a beta-ray-emitting radionuclide with a gamma ray of low probability; and ^237^Np would be the first high-atomic-number nuclide measured recently. Availability of suitable material of the first three radionuclides is being checked by section members.

### 2.2 The International Reference System (Système International de Référence, SIR) for Gamma-Ray Emitting Radionuclides

Comparisons are time-consuming activities which require a concentrated action by all participants at the same time. Obviously comparisons can only address a small fraction of the required radionuclides, even supposing that national capability for accurate measurement of a radionuclide can be maintained, once demonstrated.

Several years ago a more general comparison method was established at BIPM for gamma-ray-emitting radionuclides [5]. Samples of any suitable radionuclide are submitted at a convenient time as a specified volume of solution in uniform ampoules. The response for a stated amount of activity in two re-entrant ionization chambers is compared with that of a radium reference source, and the ratio per becquerel is registered on tables for that radionuclide containing the same information for samples from other laboratories.

At the present time 373 results for 48 radionuclides from 25 laboratories have been registered. The scheme allows not only a comparison of standards between nations, but also provides a back-up repository of measurements of a given nation with time, in case similar ionization chambers used to retain standards in the national laboratory become inoperable.

The success of the present SIR, and the demand for demonstrated international agreement for radionuclides not emitting suitable gamma rays, led to the formation of a working group at the ninth Radionuclides-Section session to investigate extension. A survey of possible users indicated that demand for three further types of desired radionuclides might be satisfied in a straightforward manner, albeit with considerable testing to determine reliability and comparison uncertainty.

Gaseous radionuclides which emit suitable gamma rays can be compared in the present chambers, once a suitable ampoule is selected. One possible ampoule is being studied.

Measurements in some member laboratories suggest that the bremsstrahlung from radionuclides emitting only beta particles with maximum energy greater than 600 keV can probably be compared with the present BIPM chambers, and this can be tested. For radionuclides emitting photons which are highly absorbed by the well walls of the present BIPM chambers, thinner-walled chambers are suggested. Members were asked to submit specifications of suitable chambers by the end of 1989.

For radionuclides emitting even lower-energy photons or only beta-particles, two possibilities were suggested. Calorimetry [6] would not require opening of sealed containers supplied by participants, but required activity levels are much greater than most laboratories wanted to supply.

Another possibility, of potentially universal application, is liquid-scintillation counting. Previous bilateral comparisons using this method involved the preparation of extensive sets of samples. However, one laboratory proposed a system using calculated response functions for each radionuclide in a system comparing the coincidences between two and three phototubes viewing the same scintillator [6]. This possibility, which might allow each laboratory to prepare sealed samples, is to be further discussed and possibly tested in a working group.

### 2.3 Other Working Group Actions

Although they cannot be discussed here in detail, several other actions involving techniques or specific topics were discussed.

#### 2.3.1 Principles of the Coincidence Methods

Several of the direct activity measurement methods [6] make use of the time relation between the initial and subsequent radiations in a decay. Nineteen drafts of papers, reports, or working party notes were circulated between the 1987 and 1989 sessions. Many were concerned with the corrections for detector pulses lost in the time a counting system is insensitive due to a previous pulse [7].

#### 2.3.2 High-Count-Rate Measurements

Some of the corrections which generate the major uncertainties in activity measurements are better tested with rates beyond those normally used. Several techniques have been developed since the last working group test, but questionnaire results suggested that other section actions should be carried out first while the best testing scheme is devised.

#### 2.3.3 ^226^Ra Standards

“Radium” is unique in that international standards, by mass, do exist. A set was prepared by Hönigschmid in 1934. Two laboratories in countries where large radium sources find extensive use suggested a repackaging of the original set from glass to metal tubes. However, great difficulty in opening a similar source prepared in 1924 showed that fears of breakage due to pressure buildup and glass deterioration are probably not warranted. Laboratories who no longer use or desire the Hönigschmid standards they have were requested to supply them to the two laboratories that use them.

#### 2.3.4 High-Efficiency NaI(T1) Techniques

If each decay of a radionuclide results in a cascade of x rays or gamma rays, a well-type NaI(T1) detector can detect at least one radiation most of the time, and the fraction lost can be calculated accurately if the efficiency for different energies is known. A report on experiences in member laboratories is to be published.

## 3. BIPM Activities

In addition to maintaining the SIR, and investigating its extension, the BIPM staff participates in the general comparisons and prepares the detailed reports describing the results. They also make fundamental contributions to the development of measuring techniques and their analysis.

Examples presented at this session were the measurement of the half life of a metastable state following the decay of ^75^Se, with a preliminary value of (16.3±0.3) ms; the development of corrections for circuit dead times in series, with possible mixtures of the two types which further extend when a second pulse arrives during a dead period, or not [8]; and possible applications of modulo-2 counting for identifying dead times.

## 4. Laboratory Reports

A final portion of this session, and all recent ones, has been the presentation of written and oral reports from each attending laboratory covering topics of possible general interest not discussed elsewhere in the agenda. These cover not only discoveries and new data, but also trends in the demands and delivery of standards to users.

## 5. Discussion

The unusual nature of radioactivity standardization makes international cooperation especially significant. The interchange of ideas about basic standardization during the sessions of the Radionuclides Section of the CCEMRI at BIPM, and about applied radioactivity measurements at associated meetings of the International Committee for Radionuclide Metrology, contributes to both the efficiency and accuracy of such measurements. The examples displayed in this article from the 10th session of the Radionuclides Section illustrate the scope and range of these activities.
